# The Effect of Time-Equated Concurrent Training Programs in Resistance-Trained Men

**DOI:** 10.5114/jhk/185637

**Published:** 2024-04-15

**Authors:** Chad Dolan, Justin M. Quiles, Jacob A. Goldsmith, Kristin M. Mendez, Alex Klemp, Zac P. Robinson, Joshua C. Pelland, Catherine Coccia, Michael C. Zourdos

**Affiliations:** 1 Muscle Physiology Laboratory, Department of Exercise Science and Health Promotion, Florida Atlantic University, Boca Raton, FL, USA.; 2 James J. Peters VA Medical Center, Spinal Cord Injury Research, Bronx, NY, USA.; 3 Department of Neurology, Icahn School of Medicine at Mount Sinai, New York, USA.; 4 Department of Dietetics and Nutrition, Florida International University, Miami, FL, USA.

**Keywords:** resistance exercise, aerobic training, strength, hypertrophy

## Abstract

The purpose of this investigation was to compare the effects of three different concurrent training (CT) programs and a resistance training (RT) program. Twenty-three resistance trained men (age: 24 ± 3 years) were randomized into four groups: concurrent RT and high intensity interval cycling (CTH, n = 6), concurrent RT and moderate intensity continuous cycling (CTM, n = 5), RT and barbell circuit training (RTC, n = 6), or RT only (RT, n = 6). Back squat and bench press strength, quadriceps, and pectoralis muscle thickness, VO_2peak_, and maximum workload (W_max_, Watts) were assessed. Squat strength gains were meaningful in all groups and comparable among CTH (16.88 kg [95% CrI: 11.15, 22.63]), CTM (25.54 kg [95% CrI: 19.24, 31.96]), RTC (17.5 kg [95% CrI: 11.66, 23.39]), and RT (20.36 kg [95% CrI: 15.29, 25.33]) groups. Bench press strength gains were meaningful in all groups and comparable among CTH (11.86 kg [95% CrI: 8.28, 15.47]), CTM (10.3 kg [95% CrI: 6.49, 14.13]), RTC (4.84 kg [95% CrI: 1.31, 8.47]), and RT (10.16 kg [95% CrI: 7.02, 13.22]) groups. Quadriceps hypertrophy was meaningful in all groups and comparable among CTH (2.29 mm [95% CrI: 0.84, 3.76]), CTM (3.41 mm [95% CrI: 1.88, 4.91]), RTC (2.6 mm [95% CrI: 1.17, 4.05]), and RT (2.83 mm [95% CrI: 1.55, 4.12]) groups. Pectoralis hypertrophy was meaningful in CTH (2.29 mm [95% CrI: −0.52, 5.1]), CTM (5.14 mm [95% CrI: 2.1, 8.15]), and RTC (7.19 mm [95% CrI: 4.26, 10.02]) groups, but not in the RT group (1 mm [95% CrI: −1.59, 3.59]); further, between-group contrasts indicated less pectoralis growth in the RT compared to the RTC group. Regarding cardiovascular outcomes, only the RTH and RTM groups experienced meaningful improvements in either measure (VO_2peak_ or W_max_). These data suggest that the interference effect on maximal strength and hypertrophy can be avoided when the aerobic training is moderate intensity cycling, high intensity cycling, or a novel barbell circuit for ~one hour per week and on non-RT days. However, the barbell circuit failed to elicit meaningful cardiovascular adaptations.

## Introduction

Concurrent training (CT) is the inclusion of both resistance (RT) and aerobic training (AT) within the same program (Hickson, 1980). Commonly, CT is used for improving body composition by weight class or physique sport athletes. Although CT improves body composition, previous data (Bell et al., 1997; Hickson, 1980; Shaw et al., 2009) have demonstrated that the inclusion of AT in a RT program can attenuate muscle strength, hypertrophy, and power adaptations, known as the interference effect (Hickson, 1980).

The interference effect can manifest both acutely and chronically during CT. Acutely, the additional fatigue from AT can decrease RT work capacity (total volume) when performed on the same day (Abernethy, 1993). Given the positive relationship between RT volume and adaptations (Ralston et al., 2017; Schoenfeld et al., 2017a), decreasing RT work capacity could impair RT benefits. Chronically, excessive AT during a RT program may compromise recovery by increasing training sessions and total stress (Goto et al., 2004; Hickson, 1980; Rhea et al., 2002). Additionally, CT promotes divergent signaling pathways (AT: ubiquitin proteasome system; RT: mammalian target of rapamycin-mTOR) (Coffey and Hawley, 2007) and neuromuscular adaptations (Bell et al., 1997; Hakkinen et al., 2003; Hickson, 1980). Specifically, AT causes fiber-type interconversions toward type I, while RT facilitates interconversions toward type II (Wilson et al. 2012a). In general, if RT adaptations are the primary goal, performing AT violates the foundational principles of specificity.

Despite the potential negative effects of the interference effect on RT adaptations, previous data have demonstrated that carefully designed programming (Wilson et al., 2012b) (i.e., intensity, duration of AT) and sufficient calorie intake (Murach and Bagley, 2016) can minimize or avoid the interference effect. For example, RT adaptations are significantly less hindered with shorter duration (i.e., 30–40 min) AT compared to longer (50–60+ min) bouts (Wilson et al., 2012b), and when separating AT bouts by at least 3 h from RT bouts (Schumann et al., 2022). Additionally, the modality of AT during CT may influence the magnitude of the interference effect, with some analyses reporting less of an interference effect from cycling compared to running (Lundberg et al., 2022; Wilson et al., 2012b) on lower body strength and hypertrophy, possibly due to less muscle damage, a lower session rating of perceived exertion (RPE), and reduced muscle soreness (Krzysztofik et al., 2023; Mathieu et al., 2022; Wilson et al., 2012b). However, other analyses report no difference between running and cycling (Sabag et al., 2018; Schumann et al., 2022). Further, high intensity interval training may aid in the attenuation of the interference effect when used as AT (Balabinis et al., 2003; Chen et al., 2024; Lee et al., 2020) due to the similarities to RT regarding cellular and neuromuscular adaptations and being consistent with the principles of specificity. Therefore, the current evidence suggests that shorter duration AT, especially high intensity interval training, performed on a separate day from lower body RT will most likely diminish interference with hypertrophy and strength during CT.

However, for athletes focused on maximizing RT adaptations during CT, there may be more optimal approaches than performing traditional AT. For example, circuit RT is effective to enhance both muscular performance and body composition (Alcaraz et al., 2008, 2011) and adheres more closely to the principle of specificity than AT. Further, if circuit RT replaced the traditionally used modes of AT during CT, total RT volume would increase, which has a positive relationship with both hypertrophy (Schoenfeld et al., 2017a) and strength (Ralston et al., 2017). Thus, it is possible that circuit RT could not only diminish the interference effect when used as a mode of AT, but could potentially enhance hypertrophy and strength adaptations to a greater degree than RT alone via an increase in total training volume.

Therefore, the primary aim of this study was to compare the effects of four different eight-week interventions in resistance trained males: (1) RT only [RT]; (2) concurrent RT and high intensity interval cycling [CTH]; (3) concurrent RT and moderate intensity continuous cycling [CTM]; (4) RT and barbell circuit training [RTC], on muscular hypertrophy and strength. It was hypothesized that RTC would elicit the greatest muscular improvements followed by RT, that CTH would have similar adaptations to RT, and CTM would produce the lowest degree of muscular improvements.

## Methods

Participants reported to the laboratory 42 times over eight and a half consecutive weeks. All CT groups (CTH, CTM, RTC) trained five days per week, while RT trained three times per week. All groups performed the same daily undulating programming RT protocol on non-consecutive days (i.e., Monday, Wednesday, Friday). The RT program featured the back squat and the bench press as main exercises, and the barbell overhead press, the barbell bent-over row, and the barbell biceps curl as accessory exercises. CT groups performed the group-specific AT modality on the days between RT sessions (i.e., Tuesday, Thursday), which was controlled for time (30 min each group). The protocol design is displayed in Table 1A.

**Table 1A:** Weekly schedule of the main training intervention.

Daily training session details					
Groups	Monday	Tuesday	Wednesday	Thursday	Friday
	Resistance Training	Aerobic Training	Resistance Training	Aerobic Training	Resistance Training
RT (*n* = 6)		n/a		n/a	
RTH (n = 6)		10 intervals cycling, 1:2 work:rest		10 intervals cycling, 1:2 work:rest	
	Main: 4 x 8 at 70%1RM		Main: 4 x 6 at 75%1RM		Main: 5 x 4+ at 80%1RM
RTM (*n* = 5)	Acc: 3 x 10 at 8RPE	30-min steady state cycling	Acc: 3 x 8 at 8RPE	30-min steady state cycling	Acc: 3 x 6 at 8RPE
RTC (*n* = 6)		30-min barbell circuit		30-min barbell circuit	

At pre- and post-study, one-repetition maximum (1RM) strength on the squat and the bench press, muscle thickness of the quadriceps and the chest, peak oxygen uptake (VO_2peak_), and the maximum workload (W_max_) were assessed. Week one served as an introductory training week, weeks two through seven were the main training program, while week eight served as a taper and post-study testing. Thirty minutes prior to each session (RT and AT), participants ingested branched chain amino acids (Xtend, Scivation, Burlington, N.C., USA) containing 3.5 g of leucine. Then, immediately after each training session, 30 g of whey protein (Scivation Whey, Scivation, Burlington, N.C., USA) were ingested. The branched chain amino acids and whey protein were provided to control nutrient timing. Both supplements were ingested in a powdered form mixed with water. Participants were asked to maintain regular use of non-ergogenic supplements and halt use of ergogenic supplements during the study.

### Participants

Twenty-five college-aged resistance trained males were recruited for this study. Participants were randomly assigned to one of the four mentioned groups: RT (*n* = 6), CTH (*n* = 6), CTM (*n* = 5), and RTC (*n* = 6). Two individuals were removed from participation, one due to minor injury (RTM group) and one because of non-compliance (RTH group). Therefore, data from 23 participants (age: 24 ± 3 years, body mass: 80.5 ± 10.2 kg, body fat content: 11.2 ± 4.0%) were included. Inclusion criteria were as follows: (1) at least two years of resistance training experience; (2) a minimum training frequency of the squat and the bench press of once per week for the previous six months immediately preceding participation; (3) a 1RM squat of ≥1.5 times body mass and a minimum bench press of ≥1.25 times body mass; (4) semi-regular consumption of whey protein during the previous six months. These criteria were confirmed via a physical activity questionnaire (Zourdos et al., 2016a). Additionally, a health history questionnaire was completed and participants were excluded if any contraindications to exercise were reported. Finally, all participants signed an informed consent form that was approved by the Florida Atlantic University Institutional Review Board (protocol code: 680161-3; approval date: 18 November 2014).

### Measures

*1RM Testing*. Testing for 1RM was performed in accordance with previously validated procedures (Zourdos et al., 2016b) following a five-minute dynamic warm-up. To find the most accurate 1RM, investigators used the average velocity (m·s^−1^) via a Tendo Weightlifting Analyzer (TENDO Sports Machines, Trencin, Slovak Republic) and participants reported their RPE (Zourdos et al., 2016b) to determine the following attempt. Each participant was given five to seven minutes of rest between 1RM attempts. A 1RM was accepted as valid if one of three conditions was met: (i) the participant reported a ‘10’ on the RPE scale and the investigator determined a subsequent attempt with increased weight would not be successfully completed, (ii) the participant reported a ‘9.5’ RPE and failed the subsequent attempt with a load increase of 2.5 kg or less, (iii) the participant reported an RPE of 9 and failed the subsequent attempt with a load increase of 5 kg or less. The squat and the bench press were performed under the rules and regulations of the United States of America Powerlifting (USAPL and Administrators, 2001).

*Wilks Score*. The Wilks score is a validated measure of relative strength (Vanderburgh and Batterham, 1999). This calculation compares strength levels of individuals with various body masses by multiplying the amount of weight lifted (i.e., 1RM) by a standardized body weight coefficient.

*Anthropometric and Relative Body Composition*. Body height (cm) was measured using a wall-mounted stadiometer and body mass (kg) was assessed via a calibrated digital scale. Body fat content or relative body composition was assessed with the BodyMetrix BX-2000 A-mode ultrasound (BodyMetrix, IntelaMetrix, Livermore, CA) and lean body mass was then calculated (Campbell et al., 2018). To assess subcutaneous fat thickness, the ultrasound probe emits a single beam with a standardized frequency of 2.5 MHz. The probe was connected by USB to a laptop loaded with the manufacturer software (BodyView Professional Software). Measurements were taken at the thigh, the chest, and the abdomen from the right side of the body, while the participant was standing. During sampling, the probe was held perpendicular to the participant with minimal movement across the skin (+/− 5 mm) and enough pressure to maintain surface contact between the device and the participant, but not depressing the participant’s subcutaneous fat tissue. Manufacturer directions were followed, and the average of two scans was used for assessment. The average represented the final site-specific subcutaneous adipose tissue thickness measurement. The software calculated body composition via Jackson and Pollock 3-site formula (Jackson and Pollock, 1978).

*Muscle Thickness*. Muscle thickness was assessed via ultrasonography (BodyMetrix Pro System, IntelaMetrix, Inc., Livermore, CA., USA). The ultrasound settings (frequency: 2.5 MHz, depth: 60 mm) were kept constant to standardize the measurements of the targeted muscles. All scans were performed on the right side of the body prior to 1RM assessment on pre- and post-testing days. The muscle at each site was scanned laterally to medially with the transducer positioned perpendicularly to the skin. Two scans were completed at each site with the average used for analysis; however, if there was a difference of >2 mm between scans, a third scan was taken. In the event of a 3^rd^ scan, the average of the two measurements within 2 mm was used. Participants were positioned supine on a massage table in an anatomical position for at least 10 min to allow for fluid compartment shifts to occur prior to the initiation of scans. The chest site was determined as half the distance between the anterior axillary line and the nipple. Three lower body sites: lateral quadriceps mid (LQM), lateral quadriceps distal (LQD), and anterior quadriceps (AQ), were identified. The LQM and LQD sites were measured at 50% and 70%, respectively, of the distance from the greater trochanter to the lateral epicondyle of the femur (Klemp et al., 2016), while AQ was assessed at 70% of the distance from the greater trochanter to the medial epicondyle of the femur. The same investigator performed palpations and scans throughout the study.

*VO_2peak_ & W_max_ Cycle Test*. Pre- and post-study VO_2peak_ testing was performed using previously validated procedures (Leveritt et al., 2003). Each participant was outfitted with a heart rate monitor (FT1 Heart Rate Monitor, Polar, Kempele, Finland), and an electronically braked cycle ergometer (Excalibur Sport, Lode, Netherlands) was used for the incremental exercise test. After a three-minute warm-up at 25 Watts (W), one-minute stages were employed, starting at 50 W, and increasing in the workload by 25 W each stage, until test termination. Participants pedaled at a fixed cadence of 80 revolutions per minute (RPM). During the test, respiratory gases were monitored and continuously analyzed by open-circuit spirometry (True One 2400+ Metabolic Measurement System, Parvo-Medics Inc., Provo, UT). The metabolic system measured minute ventilation, the oxygen consumption rate, the carbon Dioxide expiration rate, and the respiratory exchange ratio (RER). Data were averaged over 30-s intervals. The metabolic cart was calibrated prior to each test with room air for the flow rate and gases (i.e., O_2_, CO_2_) of known volume and concentration. The heart rate (HR), the workload (W), and the RPE (Borg 20-point scale) were measured and recorded at the end of every stage (last five seconds). Tests were terminated when the pedal cadence of 80 RPM could not be maintained for > 10 s or due to volitional fatigue. Tests were accepted as peak tests if participants met any two of the following criteria: plateau in VO_2_ despite an increase in the workload (<150 ml/min); RPE ≥ 17; RER > 1.15; HR ≥ 95% of age-predicted maximum (220 − age). W_max_ was calculated from the formula, W_max_ = W_f_ + (t/180) · 25, where W_f_ = the value of the last completed workload (W); t = the time the last workload was maintained (s), and 25 = the W output difference between the last two workloads (W).

### Design and Procedures

The exact details of the training program including sets, repetitions, loading progressions, and adjustments for all exercises during the study are presented in Table 1A–D.

**Table 1B:** Summary of weekly progression based on Friday “plus set.”

Week	Weekly Load	Weekly Repetition Target	Repetitions Performed	Weekly Load Adjustment
1	70–75%1RM		2	−2.5 kg
2	80%		3	+0.0 kg
3	APRE		4	+1.0 kg
4	APRE	4	5	+2.5 kg
5	APRE		6 or 7	+5.0 kg
6	APRE		8 or more	+7.5 kg
7	APRE			

**Table 1C:** Summary of load adjustments due to incomplete or failed repetitions on main exercises.

Failed Repetitions	Load Adjustment
1	−2.5 kg
2	−5.0 kg
3	−7.5 kg
4	−10.0 kg

**Table 1D:** Summary of RPE/RIR scale-based load adjustments to accessory exercises every set.

Target RPE(RIR)	Reported RPE(RIR)	Load Adjustment
	5–6(4–6)	+5.0 kg
	7(3)	+2.5 kg
8(2)	8(2)	+0.0 kg
	9(1)	−2.5 kg
	10(0)	−5.0 kg

Week 1 (introductory microcycle) consisted of 1 less set for all exercises and 5–10% lower training loads on main exercises; Main = back squat and bench press; ACC = barbell overhead press, barbell bent-over row, and barbell biceps curl; training prescription = sets x repetitions; no training occurred on Saturday and Sunday; RT = resistance training control group; RTH = high intensity interval cycling group; RTM = moderate intensity steady state cycling group; RTC = barbell circuit training group; %1RM = percentage of one repetition maximum strength; RPE = rating of perceived exertion; plus set = performance set for squat and bench press where the last set taken to volitional repetition maximum, denoted by 5 x 4+; APRE = autoregulated progressive resistance exercise; RPE/RIR = resistance training specific rating of perceived exertion scale (RPE) based on repetitions in reserve (RIR), denoted by RPE value (RIR value)

*Squat and Bench Press*. Loads were pre-planned for the introductory microcycle (week 1) and the first week of the main training cycle (week 2; Table 1A). For weeks 3–7, load progression was individualized based upon weekly performance assessment or “plus set”, which is known as autoregulatory progressive resistance exercise (Mann et al., 2010), and this load progression can be seen in Table 1B. Further, if a participant failed to complete the prescribed repetitions for any main lift set, there was a 2.5-kg reduction in the load per repetition failed on subsequent sets (Table 1C), and a 2.5-kg load reduction in that exercise for the remainder of the week. Finally, when a repetition was missed, the plus set based load progression was reduced by 50% for that exercise the following week. Load progression was resumed as planned when an entire training week was completed as prescribed. Investigator-administered rest intervals were 5–7 min for main exercises (Zourdos et al., 2016b). In the final week of training (taper microcycle; week 8), participants performed pre-planned sets, repetitions, and loads (reduced volume, but similar loads) the first two sessions of the week to prepare for their post-testing session.

*Accessory Exercises*. For accessory exercises, participants were asked to perform the repetitions at a predetermined load corresponding to a RIR-based RPE (Zourdos et al., 2016b). For the first set during week 1, participants were instructed to choose a load that would elicit an RPE of 8 (RIR = 2). In all other weeks, the final set load used in the previous week was used as the starting load of the next week. In each session, the load was increased or decreased for the subsequent set if the target RPE was not met. The details of load changes can be seen in Table 1D. Rest intervals of 1–3 min were used.

*Concurrent Training Protocols*. All CT interventions were performed on off days from RT. During weeks 1 and 8, the CT interventions were performed once per week for 18 min, and during weeks 2–7 the CT interventions were performed twice per week for 30 min.

*Concurrent Training High Intensity (CTH Group)*. The intervals consisted of 60 s of work followed by 120 s of active recovery (1:2 work:recovery). The intensity was set to 90%W_max_ for week 1, 100%W_max_ for weeks 2 and 3, 105%W_max_ for weeks 4 and 5, and 110%W_max_ for weeks 6 and 7, and 95%W_max_ for week 8. Participants were instructed to cycle as fast as possible during each work period, and to maintain slow cycling without any resistance during each recovery period.

*Concurrent Training Moderate Intensity (CTM Group)*. The continuous cycling intensity was set to the workload (W) at 30% VO_2peak_ during week 1, 40%VO_2peak_ during weeks 2 and 3, 45%VO_2peak_ during weeks 4 and 5, 50%VO_2peak_ during weeks 6 and 7, and 35% VO_2peak_ during week 8. All participants were instructed to pedal at a maintainable pace with minimal variation in RPM for the duration of each exercise session.

*Resistance Training Circuit (RTC Group)*. The resistance training circuit consisted of the same exercises from the RT program performed in a series to a prescribed number of repetitions on each exercise. Completion of one set of all exercises was considered “one round”, resulting in an average of 4.2 rounds per session. The objective was to complete as many rounds as possible in 30 min. Squat and bench press exercises were performed at 40% of 1RM and accessory exercises at 75% of the load used on the first day of each week of the RT program (i.e., Monday). The exercises were organized in the following series: a back squat, a barbell overhead press, a bench press, a barbell bent-over row, and a barbell biceps curl. All repetitions were required to be completed for each exercise before progressing to the subsequent exercise in the series. During week 1, eight repetitions were performed for all exercises, and one repetition was added bi-weekly during the main RT program (i.e., weeks 1, 2, and 3: 8 repetitions, weeks 4 and 5: 9 repetitions, weeks 6 and 7: 10 repetitions). During the taper, the load was reduced (i.e., main exercises: 35% of 1RM; accessory: 70% of the Monday’s load) and 10 repetitions were performed of each exercise.

*Dietary Recalls*. To inform dietary intake, investigators performed 24-h dietary recalls three times during the first and the final week of the study. Investigators were directly trained by a registered dietitian to perform the recalls. This was performed to educate participants regarding their nutritional habits in an effort to ensure maintenance of these habits throughout the study.

### Statistical Analysis

All analyses were conducted in R language and environment for statistical computing (v 4.3.0; R Core Team, https://www.r-project.org/). All data and code can be accessed at <https://osf.io/9t7ny/>. In addressing our research questions, we avoided dichotomizing the findings and did not employ traditional null hypothesis significance testing, which has been extensively critiqued (Amrhein et al., 2019). Instead, we took an estimation-based approach within a Bayesian framework in which all outcomes compatible with the data were considered, with the greatest emphasis placed on the point estimates using the “*brms*” and “*marginaleffects*” packages (Kruschke and Liddell, 2018; Mengersen et al., 2016).

To incorporate our expectations given previous data and to improve the precision of our estimates given a small sample size, weakly informative prior distributions were used. Specifically, the data from our lab using three similarly designed training studies (Helms et al., 2018; Klemp et al., 2016; Robinson, 2021) was used to inform the expected distributions of changes in strength and muscle size. Additionally, we consulted the best available evidence to determine the expected differences between RT and CT conditions (Schumann et al., 2022). Because our lab did not have necessary data to inform the expected distributions for changes in cardiovascular outcomes (VO_2peak_ and W_max_), these models were only fit with the default uninformed priors.

Each model used four Monte Carlo Markov Chains with 1000 warm-up and 8000 sampling iterations. Before extracting any estimates, each model was visually examined via trace plots to inspect chain convergence and posterior predictive checks to examine model validity. For the variables of interest from each model (i.e., marginal effects for condition), draws (n = 8000) were taken from the posterior distribution to construct a probability density function (i.e., mean and quartile intervals) that was used to make probabilistic inferences. The probability density functions related to the primary research questions were also compared to a region of practical equivalence (ROPE). For hypertrophy outcomes, the ROPE was defined by the typical error of measurement (Swinton et al., 2018; Weir, 2005); however, for strength and cardiovascular outcomes, the ROPE was defined as the raw-unit equivalent of a ± 0.25 standardized mean difference (Swinton et al., 2022).

To compare changes in 1RM strength (back squat and bench press exercises), pectoralis major hypertrophy, and cardiovascular fitness (VO_2peak_ and W_max_) linear regression models were constructed to mimic an analysis of covariance (i.e., ANCOVA) with an adjustment for the baseline value of the dependent variable. Specifically, change from baseline was considered the response variable while condition (4-level categorical) and the pretest value of the dependent variable (continuous) were included as population-level effects. However, for changes in quadriceps muscle size linear mixed effect models were used. Specifically, change from baseline was considered the response variable while condition (4-level categorical), site (3-level categorical), and the pretest value of the dependent variable (continuous) were included as population-level effects. As the model contained multiple observations per participant, group-level intercepts were included. After initially fitting the model with a maximal group-level slope structure (Barr et al., 2013; Oberauer, 2022), the model was reduced to include group-level slopes for the site and the pretest value of the dependent variable at the participant level.

## Results

Descriptive summaries (i.e., mean ± standard deviation) of participants’ characteristics can be seen in Table 2. All model output and the unadjusted values of the primary outcomes can be found in the supplementary materials <https://osf.io/9t7ny/>.

**Table 2:** Descriptive Summaries.

Characteristic	RT (n = 6)	RTC (n = 6)	CTM (n = 5)	CTH (n = 6)
Age (years)	23.67 ± 4.27	22.33 ± 1.75	24.80 ± 2.28	24.33 ± 2.94
Height (cm)	174.09 ± 5.47	175.90 ± 6.09	176.25 ± 8.14	175.13 ± 8.88
Pre Body Mass (kg)	79.07 ± 5.81	78.60 ± 8.71	78.29 ± 15.68	85.53 ± 9.79
Post Body Mass (kg)	80.72 ± 6.66	80.71 ± 6.80	79.10 ± 16.37	86.99 ± 10.43
Δ Body Mass (kg)	1.66 ± 3.22	2.11 ± 2.85	0.81 ± 2.38	1.47 ± 1.28
Pre Estimated Body Fat (%)	10.90 ± 2.66	11.30 ± 2.83	9.38 ± 2.22	13.10 ± 6.50
Post Estimated Body Fat (%)	11.63 ± 4.18	12.80 ± 3.15	11.08 ± 3.83	14.02 ± 5.72
Δ Estimated Body Fat (%)	0.73 ± 4.63	1.50 ± 2.25	1.70 ± 2.15	0.92 ± 2.77
Pre SQ + BP Wilks (a.u.)	176.85 ± 21.59	175.83 ± 22.99	166.13 ± 18.39	169.79 ± 26.14
Post SQ + BP Wilks (a.u.)	196.61 ± 21.14	186.61 ± 21.52	191.87 ± 19.30	184.92 ± 24.57
Δ SQ + BP Wilks (a.u.)	19.76 ± 9.36	10.78 ± 5.23	25.73 ± 3.84	15.13 ± 8.19

SQ = Squat; BP = Bench Press

### Back Squat 1RM

**Figure 1:**
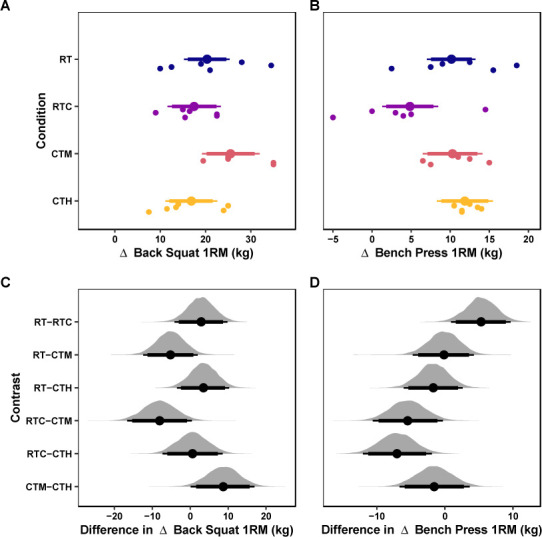
Strength Outcomes. *Marginal posterior distributions for changes in the back squat 1RM (A) and the bench press 1RM (B) and differences among conditions for the back squat 1RM (C) and the bench press 1RM (D). Vertical dashed lines represent the region of practical equivalence (i.e., ROPE) defined by the raw-unit equivalent of a ± 0.25 standardized mean difference. Colored dots and intervals represent the mean and quartile intervals (90 and 95%) from the posterior distribution. Finally, individual data are visualized below with solid* circles. *The marginal effects are adjusted for the pretest scores of the dependent variable*.

The mean values of the marginal posterior distributions suggest that all conditions demonstrated meaningful increases in back squat 1RM strength. Specifically, the RT condition presented an increase of 20.36 kg [95% CrI: 15.29, 25.33] with a 100% probability of the change being greater than the ROPE. The RTC condition presented an increase of 17.5 kg [95% CrI: 11.66, 23.39] with a 100% probability of the change being greater than the ROPE. The CTM group observed an increase of 25.54 kg [95% CrI: 19.24, 31.96] with a 100% probability of the change being greater than the ROPE. Finally, the CTH group observed an increase of 16.88 kg [95% CrI: 11.15, 22.63] with a 100% probability of the change being greater than the ROPE. The credible intervals of all contrasts among conditions were compatible with the ROPE (Table 3). These results are visualized in Figure 1A&C.

**Table 3:** Contrasts of Marginal Effects.

		95% Credible Interval		90% Credible Interval		
Contrast	Mean	Lower Bound	Upper Bound	Lower Bound	Upper Bound	Probability > ROPE
*Back Squat 1RM (kg)*						
RT-RTC	2.8609334	−4.0846545	9.8445959	−2.9900589	8.6733361	19.09
RT-CTM	−5.1817953	−12.3641477	1.9650008	−11.2418077	0.8447211	41.65
RT-CTH	3.4811883	−3.4673020	10.2776647	−2.3851331	9.1622511	24.33
RTC-CTM	−8.0427287	−16.5812854	0.4734758	−15.2711040	−0.8034491	68.94
RTC-CTH	0.6202549	−7.2826118	8.5589916	−5.9941269	7.3057987	9.79
CTM-CTH	8.6629836	0.1481244	16.9454710	1.5896963	15.6504887	73.94
*Bench Press 1RM (kg)*						
RT-RTC	5.3158512	0.8971960	9.6453147	1.6036098	8.9430146	68.79
RT-CTM	−0.1416484	−4.7170248	4.2473153	−3.9035598	3.5351813	3.67
RT-CTH	−1.7025270	−6.0350349	2.6154737	−5.3529359	1.9390903	12.30
RTC-CTM	−5.4574996	−10.5828831	−0.3042842	−9.7186019	−1.1019246	67.45
RTC-CTH	−7.0183782	−12.0280629	−1.9001775	−11.2486451	−2.7617697	85.88
CTM-CTH	−1.5608787	−6.6396648	3.6380486	−5.8887099	2.7970995	15.08
*Quadriceps MT (mm)*						
RT-RTC	0.2331128	−1.0231289	1.4824821	−0.8196912	1.2758891	18.77
RT-CTM	−0.5715763	−1.8668436	0.7520933	−1.6625213	0.5313926	37.55
RT-CTH	0.5477428	−0.7351115	1.7857279	−0.5153661	1.5828387	35.30
RTC-CTM	−0.8046890	−2.4362611	0.8760444	−2.1592544	0.5925250	51.15
RTC-CTH	0.3146300	−1.2972764	1.8950979	−1.0328927	1.6396616	27.81
CTM-CTH	1.1193190	−0.6436698	2.7594095	−0.3361734	2.4979418	65.63
*Pectoralis Major MT (mm)*						
RT-RTC	−6.1938923	−9.7729415	−2.4186233	−9.2605426	−3.0545362	98.55
RT-CTM	−4.1446791	−7.8649111	−0.3213127	−7.2288202	−0.9459705	87.24
RT-CTH	−1.2934901	−4.9269436	2.3404023	−4.2923877	1.7241561	36.23
RTC-CTM	2.0492132	−2.1260678	6.1317030	−1.4460896	5.5172007	52.23
RTC-CTH	4.9004022	0.8176847	8.8656350	1.4804937	8.2529682	92.28
CTM-CTH	2.8511890	−1.3134206	6.9280953	−0.5855341	6.2845286	67.12
*VO_2 peak_ (ml•kg•min^-1^)*						
RT-RTC	1.4886938	−3.5743158	6.5461344	−2.6898642	5.7195229	51.69
RT-CTM	−2.5416357	−7.7878821	2.7533342	−6.8804397	1.8936094	67.83
RT-CTH	−4.6669771	−9.8563058	0.5577217	−8.9575569	−0.4198616	89.89
RTC-CTM	−4.0303295	−9.3033274	1.2196273	−8.3330765	0.3219204	84.79
RTC-CTH	−6.1556709	−11.5342907	−0.8106426	−10.5421846	−1.7344040	96.28
CTM-CTH	−2.1253413	−7.3168312	3.1365400	−6.4149551	2.2027414	61.84
*Maximum Workload (W)*						
RT-RTC	−8.3497351	−36.0545846	19.0359073	−31.2202500	13.9357210	47.31
RT-CTM	−34.0883236	−63.3308357	−4.3644052	−58.1133701	−9.7243947	95.30
RT-CTH	−41.3920198	−68.4819356	−14.2133650	−63.7696172	−18.8117681	98.84
RTC-CTM	−25.7385884	−53.4337318	1.7631891	−48.6445671	−2.7409799	88.92
RTC-CTH	−33.0422846	−59.5051235	−6.8155657	−54.5621033	−11.3338846	96.33
CTM-CTH	−7.3036962	−35.0153318	20.8236505	−30.1572542	15.8893172	44.49

1RM = one-repetition maximum; RT = resistance training; MT = muscle thickness; RTC = resistance training circuit; CTM = concurrent training moderate intensity; CTH = concurrent training high intensity; VO_2peak_ = peak oxygen uptake

### Bench Press 1RM

The mean values of the marginal posterior distributions suggest that most conditions demonstrated significant increases in bench press 1RM strength. Specifically, the RT group observed an increase of 10.16 kg [95% CrI: 7.02, 13.22] with a 99.98% probability of the change being greater than the ROPE. The RTC group observed an increase of 4.84 kg [95% CrI: 1.31, 8.47] with a 62% probability of the change being greater than the ROPE. The CTM group observed an increase of 10.3 kg [95% CrI: 6.49, 14.13] with a 99.92% probability of the change being greater than the ROPE. Finally, the CTH group observed an increase of 11.86 kg [95% CrI: 8.28, 15.47] with a 100% probability of the change being greater than the ROPE. The credible intervals of all contrasts among conditions were compatible with the ROPE (Table 3). These results are visualized in Figure 1B&D.

### Quadriceps Hypertrophy

**Figure 2:**
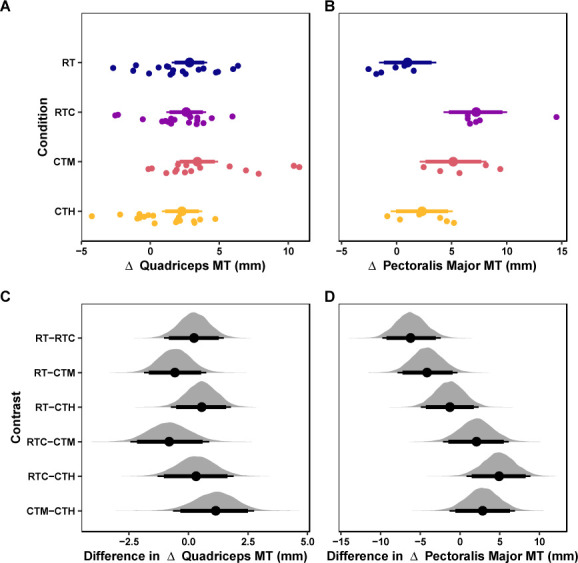
Hypertrophy Outcomes. *Marginal posterior distributions for changes in quadriceps (A) and pectoralis major muscle thickness (B) and differences among conditions for quadriceps (C) and pectoralis major muscle thickness (D). Vertical dashed lines represent the region of practical equivalence (i.e., ROPE) defined by the typical error of measurement. Colored dots and intervals represent the mean and quartile intervals (90 and 95%) from the posterior distribution. Finally, individual data are visualized below with solid circles. The marginal effects are adjusted for the pretest scores of the dependent variable, and the measurement site*.

The mean values of the marginal posterior distributions suggest that all conditions induced meaningful increases in quadriceps muscle thickness. Specifically, the RT group observed an increase of 2.83 mm [95% CrI: 1.55, 4.12] with a 99.88% probability of the change being greater than the ROPE. The RTC condition resulted in an increase of 2.6 mm [95% CrI: 1.17, 4.05] with a 99.41% probability of the change being greater than the ROPE. The CTM group presented an increase of 3.41 mm [95% CrI: 1.88, 4.91] with a 99.94% probability of the change being greater than the ROPE. Finally, the CTH group observed an increase of 2.29 mm [95% CrI: 0.84, 3.76] with a 97.86% probability of the change being greater than the ROPE. The credible intervals of all contrasts among conditions were compatible with the ROPE (Table 3). These results are visualized in Figure 2A&C.

**Figure 3:**
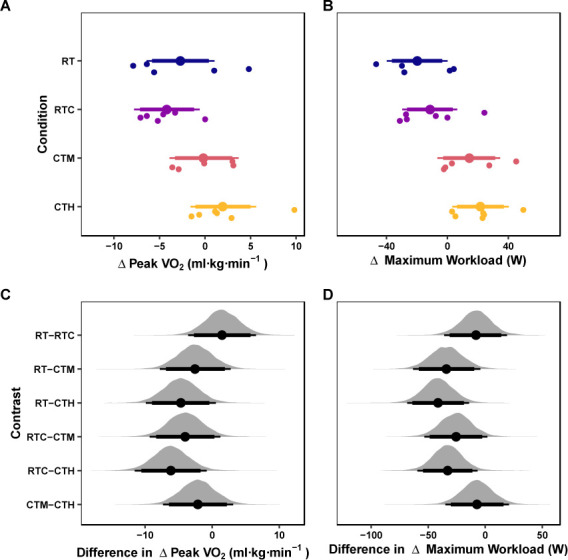
Cardiovascular Outcomes. *Marginal posterior distributions for changes in VO_2peak_ (A) and W_max_ (B) and differences among conditions for VO_2peak_ (A) and W_max_ (B). Vertical dashed lines represent the region of practical equivalence (i.e., ROPE) defined by the raw-unit equivalent of a ± 0.25 standardized mean difference. Colored dots and intervals represent the mean and quartile intervals (90 and 95%) from the posterior distribution. Finally, individual data are visualized below with solid circles. The marginal effects are adjusted for the pretest scores of the dependent variable*.

### Pectoralis Major Hypertrophy

The mean values of the marginal posterior distributions suggest that some conditions demonstrated significant increases in quadriceps muscle thickness. Specifically, the RT group observed an increase of 1 mm [95% CrI: −1.59, 3.59] with a 23.84% probability of the change being greater than the ROPE. The RTC group presented an increase of 7.19 mm [95% CrI: 4.26, 10.02] with a 99.98% probability of the change being greater than the ROPE. The CTM group observed an increase of 5.14 mm [95% CrI: 2.1, 8.15] with a 98.09% probability of the change being greater than the ROPE. Finally, the CTH group presented an increase of 2.29 mm [95% CrI: −0.52, 5.1] with a 59.58% probability of the change being greater than the ROPE. The credible intervals of all contrasts among conditions but one (i.e., RT-RTC) were compatible with the ROPE (Table 3). These results are visualized in Figure 2B&D.

### VO_2__p__eak_

The mean values of the marginal posterior distributions suggest that some conditions demonstrated meaningful changes in VO_2_. Specifically, the RT condition resulted in a change of −2.72 (ml·kg·min^−1^) [95% CrI: −6.41, 1.04] with a 77.28% probability of the change being greater than the ROPE. The RTC condition observed a change of −4.21 (ml·kg·min^−1^) [95% CrI: −7.78, −0.6] with a 94.36% probability of the change being greater than the ROPE. The CTM condition presented a change of −0.18 (ml·kg·min^−1^) [95% CrI: −3.9, 3.69] with a 26.27% probability of the change being greater than the ROPE. Finally, the CTH condition resulted in a change of 1.95 (ml·kg·min^−1^) [95% CrI: −1.6, 5.6] with a 63.02% probability of the change being greater than the ROPE. The credible intervals of all contrasts among conditions were compatible with the ROPE (Table 3). These results are visualized in Figure 3AC.

### Maximum Workload

The mean values of the marginal posterior distributions suggest that some conditions demonstrated significant changes in W_max_. Specifically, the RT condition resulted in a change of −19.8 (W) [95% CrI: −39.79, 0.06] with a 86.12% probability of the change being greater than the ROPE. The RTC condition presented a change of −11.45 (W) [95% CrI: −29.62, 6.45] with a 60.55% probability of the change being greater than the ROPE. The CTM condition observed a change of 14.29 (W) [95% CrI: −6.6, 34.65] with a 69.47% probability of the change being greater than the ROPE. Finally, the CTH condition resulted in a change of 21.59 (W) [95% CrI: 3.34, 40.35] with a 91.26% probability of the change being greater than the ROPE. The credible intervals of all contrasts but one (i.e., RT-CTH) among conditions were compatible with the ROPE (Table 3). These results are visualized in Figure 3BD.

## Discussion

The main findings of this study do not align with our hypothesis and are as follows: 1) all groups experienced meaningful increases in squat and bench press 1RM strength, with no significant between-group contrasts; 2) all groups experienced meaningful increases in quadriceps muscle thickness, with no significant between-group contrasts; 3) all groups, with the exception of the RT group, experienced meaningful increases in pectoralis major muscle thickness, with a significant between-group contrast indicating RTC > RT; 4) only the CTH group significantly increased VO_2peak_ while RT and RTC groups experienced a meaningful decrease in VO_2peak_, but no between-group contrasts were significant; 5) W_max_ meaningfully increased in the CTM and CTH groups while the RT and RTC groups experienced meaningful decreases, with a significant between-group contrast indicating CTH > RT. Overall, these results suggest that the interference effect can be avoided when the duration of AT is limited to 30 min and separated from RT by 24 h. Further, RTC does not enhance strength adaptations of RT, but may provide a slight hypertrophic benefit in the upper body.

The present study did not find evidence of the interference effect on lower body strength gains in either cycling condition (CTM or CTH). This conflicts with a recent meta-analysis (Chen et al., 2024) that reported a meaningful interference effect on lower body strength gains when AT is moderate intensity continuous cycling (SMD = −0.38; 95% CI = −0.62 to −0.14) or, to a lesser degree, high intensity interval cycling training (SMD = −0.18; 95% CI = −0.49 to 0.13). However, those authors noted a limitation of their analysis which was that 67.5% of the included studies implemented AT and RT in the same session. Indeed, another meta-analysis (Petré et al., 2021) reported the interference effect on strength gains was present in trained individuals, but not in untrained individuals; however, this was only the case when AT was performed in the same session (SMD = −0.66; −1.08 to −0.25), but not when the sessions were separated (SMD = −0.10; 95% CI = −0.43 to −0.23).

Similarly, the present study did not find evidence of the interference effect for either cycling intensity (CTH or CTM) on upper body strength gains, which aligns with a previous meta-analysis reporting no influence of lower body AT on upper body strength gains (Sabag et al., 2018). However, the RTC group experienced the smallest nominal bench press 1RM gain (4.84 kg) and lowest probability of exceeding the ROPE (62%), while all other groups experienced gains > 10 kg and a > 99% probability. While our low statistical power due to a small sample size must be considered, the present study is novel in its use of upper body AT via the barbell circuit. Thus, it is possible that the interference effect attenuated gains due to upper body AT, especially given the exercises in the barbell circuit were primarily upper body (a bench press, a barbell overhead press, a barbell bent-over row, a barbell biceps curl). Despite the low loads used in the barbell circuit, it is also possible that the minimal rest used and multiple rounds (average of 4.2 per session) led to repetitions closer to failure, potentially contributing to upper body fatigue for the subsequent RT sessions. Indeed, appropriately managing fatigue has previously been reported to enhance training performance (i.e., training loads) and subsequently 1RM strength throughout a RT program (Zourdos et al., 2016a); thus, the potential additional upper body fatigue in the RTC group may have compromised progression given the current study utilized a performance-based progression. However, caution is warranted given the low sample size, and future research is warranted to explore this question.

While the RTC group experienced the smallest increase in bench press 1RM, this group simultaneously experienced the largest nominal increase in pectoralis muscle thickness of 7.19 mm [95% CrI: 4.26, 10.02]. Thus, the additional bench press repetitions performed in the barbell circuit sessions may have provided a minor hypertrophic stimulus. Although circuit training was low load, participants subjectively indicated the protocol to be difficult, and it is plausible that acute fatigue may have led to a meaningful hypertrophic stimulus. This aligns with research indicating a dose-response relationship between volume and hypertrophy (Baz-Valle et al., 2022; Schoenfeld et al., 2017a) and that hypertrophy can be achieved with a wide loading range (Schoenfeld et al., 2017b). On the other hand, strength gains appear to have a dose-response relationship with loads (Lopez et al., 2021); thus, fatigue from the barbell circuit in the RTC group may have compromised performance and thus loads used. However, caution is once again warranted given the small sample and lack of significant between-group contrasts between RTC and all other groups.

The upper body findings are slightly opposed to the findings for quadriceps hypertrophy, in which all groups experienced relatively similar increases in muscle thickness (2.29 to 3.41 mm), with no meaningful between-group contrasts. This aligns with multiple meta-analyses that either report that lower body hypertrophy does not suffer from the interference effect (Chen et al., 2024; Sabag et al., 2018; Schumann et al., 2022), or if it does, it is diminished if AT is performed in a separate session (Petré et al., 2021) and when the duration and frequency are lower (Wilson et al., 2012b). Thus, our data provide additional evidence that hypertrophy has low likelihood of being interfered as a result of CT with certain program design choices.

However, it should be noted that while CT groups (CTM, CTH, RTC) were time-matched, the RT group had two less sessions per week. Thus, while the addition of AT generally did not lead to a meaningful *net* effect on strength and hypertrophy, it should be considered that AT can contribute to hypertrophy and strength (Ozaki et al., 2015); thus, it may be that the combined effect of RT + additional AT counteracted the interference effect in the present study.

While the barbell circuit did not introduce a clear interference effect on strength and hypertrophy adaptations, it did fail to promote cardiovascular adaptations as measured by VO_2peak_ and W_max_. Thus, the present study suggests that VO_2peak_ and W_max_ are better enhanced with traditional cardiovascular training compared to a barbell circuit in resistance trained participants. This is notable because subjective participant feedback indicated the barbell circuit was challenging. However, caution is once again warranted given the small sample size and lack of meaningful between-group contrasts.

It is suggested that CT program design decisions be made based on the desired physiological outcomes, time available to train, and sound fatigue management. It appears that some cardiovascular adaptations can occur from CTM and CTH, but the effects in the present study were modest. While ~one hour per week of AT can promote some cardiovascular adaptations and generally avoid the interference effect, a greater dosage of AT may be required to maximize cardiovascular adaptations.

The chief limitation of this study is the low sample size. Additionally, as noted, the interference effect appears to be modulated by various factors such as AT proximity to RT, AT modality, AT duration, AT frequency, training status, and nutritional energy balance. Thus, the present findings cannot be extrapolated to other configurations of program design variables or trainee nutritional energy balance. In the present study, each group experienced an average increase in body mass, indicating a positive energy balance. Indeed, a positive energy balance seems to compensate for the increased training demands of CT and has been shown to counteract the interference effect (Murach and Bagley, 2016). Therefore, since individuals in this study were not in a negative energy balance, our results cannot be extrapolated to athletes who may be in a negative energy balance, such as weight class or physique sport athletes. In other words, those in a negative energy balance should still be mindful of the potential interference effect associated with CT. Despite these limitations, the present study employed an ecologically valid CT protocol for individuals interested in maximizing RT adaptations in periods of positive energy balance.

## Conclusions

In summary, our data indicate that the interference effect on maximal strength and hypertrophy can be avoided when AT is moderate or high intensity cycling for ~one hour per week and on non-RT days. Further, the novel AT barbell circuit utilized did not promote robust cardiovascular or strength adaptations, but may be sufficient to provide a small additional upper body hypertrophic stimulus.
